# Implementation of legal abortion in Nepal: a model for rapid scale-up of high-quality care

**DOI:** 10.1186/1742-4755-9-7

**Published:** 2012-04-04

**Authors:** Ghazaleh Samandari, Merrill Wolf, Indira Basnett, Alyson Hyman, Kathryn Andersen

**Affiliations:** 1 Ipas, PO Box 5027, Chapel Hill, NC, USA; 2 Ipas Nepal, P.O. Box No. 11621, Kathmandu, Nepal

**Keywords:** Induced abortion, Reproductive health, Postabortion complications, Nepal

## Abstract

Unsafe abortion's significant contribution to maternal mortality and morbidity was a critical factor leading to liberalization of Nepal's restrictive abortion law in 2002. Careful, comprehensive planning among a range of multisectoral stakeholders, led by Nepal's Ministry of Health and Population, enabled the country subsequently to introduce and scale up safe abortion services in a remarkably short timeframe. This paper examines factors that contributed to rapid, successful implementation of legal abortion in this mountainous republic, including deliberate attention to the key areas of policy, health system capacity, equipment and supplies, and information dissemination. Important elements of this successful model of scaling up safe legal abortion include: the pre-existence of postabortion care services, through which health-care providers were already familiar with the main clinical technique for safe abortion; government leadership in coordinating complementary contributions from a wide range of public- and private-sector actors; reliance on public-health evidence in formulating policies governing abortion provision, which led to the embrace of medical abortion and authorization of midlevel providers as key strategies for decentralizing care; and integration of abortion care into existing Safe Motherhood and the broader health system. While challenges remain in ensuring that all Nepali women can readily exercise their legal right to early pregnancy termination, the national safe abortion program has already yielded strong positive results. Nepal's experience making high-quality abortion care widely accessible in a short period of time offers important lessons for other countries seeking to reduce maternal mortality and morbidity from unsafe abortion and to achieve Millennium Development Goals.

## Introduction

Nepal is a poor country whose rugged terrain and political unrest exacerbate the significant challenges of providing health care to its population of about 28 million. Until recently, Nepal reported one of the highest maternal mortality ratios in the world, with a significant proportion of maternal deaths and injuries attributable to unsafe abortion. In March 2002, responding to public health and human rights imperatives, the Nepali Parliament passed landmark legislation to reverse its archaic abortion law. For the first time in the country's modern history, the government granted women legal access to abortion. Under the new policy, which went into effect in 2003, women are permitted abortion for up to 12 weeks of gestation on request and under certain medical/legal conditions thereafter (see below) [1].

### Specifications of Nepal's 2002 Abortion Law

Pregnancy termination is available under these circumstances:

• Up to 12 weeks gestation for any indication, by request

• Up to 18 weeks gestation in the case of rape or incest

• At any time during pregnancy if mental/physical health or life of the pregnant woman is at risk (approval from a medical practitioner required)

• At any time during pregnancy if the fetus is deformed and incompatible with life (approval from a medical practitioner required)

Additional considerations:

• Only providers certified in safe abortion care are eligible to provide induced abortion services;

• The pregnant woman alone has the right to choose to continue or discontinue pregnancy

• In the case of minors (< 16 yrs of age) or mental incompetence, a legal guardian must give consent

• Pregnancy termination on the basis of sex selection is prohibited

This significant policy change followed an intensive period of advocacy and rigorous planning for implementation of safe legal abortion services. The scope and speed of abortion services scale-up in Nepal--in an extremely challenging geographic, political and economic environment--engaged multiple stakeholders from a variety of sectors in a well-coordinated, collaborative effort. Nepal's experience serves as a useful model for introduction and rapid development of safe abortion infrastructure following liberalization of abortion policy.

This article highlights key elements of planning and implementation of safe abortion care in Nepal to suggest lessons related to both successes and challenges that can be applied in other contexts to reduce maternal deaths and injuries from unsafe abortion. Perhaps the element most critical to the successful roll-out of safe legal abortion in Nepal is reliance on public-health evidence as a basis for policy, which led to the embrace of medical abortion technology and the involvement of midlevel providers as key strategies for making safe abortion care widely accessible to women. Integration of safe abortion into the country's ongoing Safe Motherhood program and into the broader health system has also been important. The authors hope that sharing the successful experience of safe abortion service implementation in Nepal can lead to improvement in women's health and contribute to achievement of health-related Millennium Development Goals in a variety of settings.

## Background

Nepal's 1854 legal code known as the Muluki Ain, which was revised numerous times until a final iteration passed in 1963, banned abortion except when the woman's life was at risk [2]. In all other cases, the edict equated pregnancy termination with homicide, and Nepal was one of the rare countries to prosecute and send women to prison under charges of infanticide [3]. Up to one-fifth of women in Nepali prisons before 2002 were convicted on the basis of illegal abortion, with many branded as murderers [4].

The negative health effects of Nepal's abortion ban were widespread and well-documented. By 1994, the abortion rate in Nepal was estimated at 117 per 100,000 women; all abortions were clandestine and many were unsafe [5]. This reliance on abortion occurred in the context of high fertility and low contraceptive use: in 1996, women had a total fertility rate of 4.63, only 29% of married women ages 15-49 reported using a contraceptive, and 31% expressed an unmet need for family planning [6]. In the period just before legal reform, Nepal's maternal mortality ratio was 539 deaths per 100,000 live births [6], with a large proportion of deaths attributed to unsafe abortion. One facility-based study found that 20% of maternal deaths were due to illegal abortion [7]. Abortion-related morbidity was also high; one hospital-based study of obstetric complications found that 53.7% of admissions were attributable to clandestine abortion [8].

In the late 1980s, with support from the United States Agency for International Development (USAID) and technical assistance from the international non-governmental organization (NGO) JHPIEGO, the Ministry of Health and Population (MOHP) began improving the quality and availability of postabortion care (emergency treatment of complications of unsafe abortion linked to postabortion contraception and other reproductive health services). Over time, growing awareness of the negative impact of unsafe abortion on women's health and lives, and of access to safe abortion as fundamental to women's rights and maternal health goals, fostered multi-sectoral support for reform of Nepal's restrictive abortion law. Advocacy efforts, led by the MOHP and well-documented elsewhere [9], culminated in 2002 with passage of the Muluki Ain 11^th ^Amendment Bill [10], a gender equality bill containing language liberalizing access to abortion.

**Figure 1:**
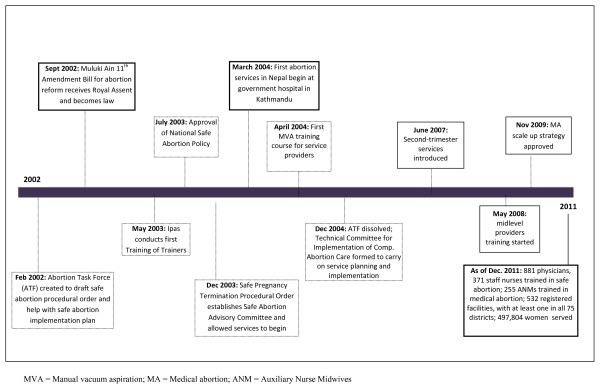
**Timeline of Strategic Steps in Safe Abortion Service Planning and Implementation in Nepal since Legal Reform in 2002**.

To guide implementation of the law, in February 2002, the MOHP's Family Health Division (FHD) created the Abortion Task Force (ATF), comprising the Nepal Society of Gynaecology & Obstetricians (NESOG), the Centre for Research on Environment Health and Population Activities (CREHPA), German Technical Assistance (GTZ) and Ipas [11]. Many organizations and individuals on the task force had also been involved in advocacy for legal reform, as well as in Safe Motherhood efforts led by Options and funded primarily by the U.K. Department for International Development (DFID). Figure 1 highlights key events in the implementation of the new law, beginning with the creation of the ATF.

The first of the ATF's two principal tasks was to draft a National Safe Abortion Policy describing strategies for preventing unsafe abortion by increasing access to safe abortion services. The ATF reviewed evidence on abortion services implementation in countries such as Vietnam and India, including traveling to Vietnam to observe delivery of comprehensive abortion care (CAC) services there. Task force working groups assessed needs and developed action plans in the four key areas of **service provision; training; monitoring and evaluation (M&E); and information, education and communication (IEC) **[11]. Approved in July 2003, the evidence-based National Safe Abortion Policy lays out the rationale for providing safe abortion and explicitly contextualizes safe abortion within Safe Motherhood efforts.

The ATF's second task was to assist the government in translating the impending legislation into a legal procedural order defining legislative mechanisms, clinical norms, regulations, roles and responsibilities for nationwide implementation of abortion care, approval of which was required before services could begin [9]. Consulting with national lawyers and international experts, the ATF incorporated technical and policy guidance on abortion care from the World Health Organization [12] to develop the Safe Pregnancy Termination Procedural Order. After multiple reviews and revisions by stakeholders, it was approved in December 2003 [13].

The government then replaced the ATF with the Technical Committee for Implementation of Comprehensive Abortion Care (TCIC), a coordinating body within the Family Health Division. Government members are the National Health Training Center, Logistics Management Division and the National Health Information, Education and Communication Center; civil-society participants are the Nepal Health Sector Support Program (a DFID-funded project), Marie Stopes International, the Family Planning Association of Nepal (FPAN), Forum for Women's Law and Development, Safe Motherhood National Federation, PSI, CREPHA and Ipas. The procedural order also created the Safe Abortion Advisory Committee (SAAC), with members from multiple government ministries, professional councils, NGOs and advocacy organizations, to oversee the TCIC and provide high-level policy guidance and program decision-making [13]. The SAAC consults closely with the national ob/gyn association, which provides recommendations to policy-makers and keeps partners abreast of advances in safe abortion care [14].

### Partnering for rapid safe abortion service implementation

Diligent planning and coordination by the groups noted above ensured that safe legal abortion services began in Nepal immediately after approval of the procedural order, followed by phased expansion. National scale-up has involved deliberate attention to the essential elements of **policy, health system capacity, equipment and supplies, and information**.

#### Policy

The procedural order laid the foundation for safe abortion service delivery by codifying decisions on such critical issues as **facility certification**, **clinical procedures, provider eligibility **and **service fees**. It designated a simple standard for **authorizing public-sector abortion care facilities**, whereby facilities are authorized, or "listed," when at least one provider is trained in safe abortion and minimum resource requirements are met (for example, availability of appropriate equipment, supplies and personnel). Private clinics must meet the same standards but receive additional auditing from the district public health office or the FHD [13]. Feedback regularly collected from partners, providers and managers is used to make the certification process more robust while maintaining simplicity and decentralization.

Regarding **clinical techniques**, the procedural order called for use of manual vacuum aspiration (MVA) and introduction of medical abortion (MA) for first-trimester abortion. Familiarity with MVA among physicians and nurses already offering postabortion care facilitated rapid expansion of induced abortion services, while the decision to authorize use of MA - pending successful pilot studies--was pivotal in decentralizing care. The procedural order focused on first-trimester services. Introduction of training and service delivery for second-trimester abortion, which involves more complex clinical procedures than first-trimester care, was intentionally delayed to allow health-care workers to develop strong first-trimester skills first. Drawing on experience in other countries showing that second-trimester abortion care can be more emotionally challenging for some providers, program leaders also recognized the need to ensure that the health workforce was well-sensitized to the issue of abortion and to women's needs before introducing these services. In 2006, a national facility-based survey found that 13% of women seeking abortion were turned away because they were more than 12 weeks pregnant [15]. With this evidence of the strong need for second-trimester care, advocates and policymakers developed a Strategic Plan for Second-Trimester Abortion based on global experience and evidence. It provided clinical standards for dilatation and evacuation and medical induction abortion and specified facility eligibility requirements (namely, hospitals with emergency obstetric care services). The MOHP's formal endorsement of the plan in April 2007 led to implementation of second-trimester services [16].

In terms of **provider eligibility**, the procedural order authorized physicians and health workers to provide abortions. Subsequently, at the request of the TCIC, the Safe Abortion Advisory Committee stipulated that health workers undergo a minimum of 12 months' pre-service midwifery training, be registered by their respective professional councils, and attend in-service training on postabortion care, insertion of intrauterine contraceptive devices or skilled birth attendance. The only midlevel providers who meet these criteria in Nepal are staff nurses and auxiliary nurse midwives (ANMs), many of whom already had experience using MVA for postabortion care. By authorizing these cadres to provide abortion, the government facilitated rapid decentralization of services to rural and remote areas. In contrast, the Strategic Plan for Second-Trimester Abortion dictated the exclusive training of obstetrician-gynecologists and general practitioners for that service, restricting it to higher-level facilities.

The **fee structure **established for public-sector maternal health services set a standard fee of about 1,000 rupees (~$14) for induced abortion. Although modest, this fee was in contrast to free provision of postabortion care, delivery, pre- and post-natal care, and long-acting contraceptives. Policymakers reportedly have been concerned that including abortion in this package of free maternal health services might promote its use as a method of contraception. The safe abortion policy does stipulate that services be provided free of charge to poor or otherwise marginalized women, but eligibility requirements have not been clearly defined and are not applied in a uniform manner, posing considerable barriers to access for vulnerable women. Women's health advocates view the discriminatory exclusion of abortion from free maternal health services as a significant barrier and are encouraging the government to revise the policy, at least for the poorest women.

Coordination with the **private sector **has also been essential in implementing safe abortion in Nepal. The principal private-sector sources of abortion care are MSI Nepal and the Family Planning Association of Nepal; services are also available at some private hospitals, clinics and individual medical practices. Private-sector providers fill an important niche in urban areas, while the public health system emphasizes serving poor women and those in rural areas (about 86% of the population according to government statistics for 2010). Private providers also refer women requiring second-trimester services or treatment of complications to government facilities. A public opinion poll conducted by CREHPA in 2006 [17] found that about three-quarters of women surveyed would prefer to visit a government hospital than other facilities for safe abortion services, citing lower cost as the main factor but also indicating a high level of confidence in the quality of care at public facilities; these findings were later supported by a 2010 survey of abortion clients at Kathmandu's Maternity Hospital and MSI's Chabel Chucchepati Clinic [18,19].

#### Health system capacity

Under the leadership of the Family Health Division, the TCIC developed systematic, comprehensive strategies for rolling out safe abortion services, including **training **in all recommended methods, **supervision **and **monitoring**. Careful attention to these elements helped ensure that the capacity of the public health system was equal to the task of providing safe legal abortion on a national scale:

The efficient and comprehensive **training **of service providers in safe abortion, beginning well in advance of service initiation, was a cornerstone of rapid expansion. As part of preliminary planning, the TCIC training sub-group, with assistance from Ipas, developed safe abortion reference and training manuals and a curriculum initially focused on competency-based training on MVA [20]. The curriculum includes instruction in technical abortion procedures, counseling, postabortion contraception and guidance for improved facility functionality.

In May 2003, before the procedural order was even approved, 20 senior gynecologists from central/regional hospitals and NGO/private clinics with previous training experience were initiated in a Training of Trainers course in Kathmandu. These providers (specifically those at the Maternity Hospital in Kathmandu) were the first to offer legal abortion services, beginning in March 2004. They began instructing additional physicians (from both the public and private sectors) recruited for training by Ipas and TCIC. This cascade training initially focused only on MVA and was sequenced to foster rapid national scale-up. The first rounds included providers from all regional and zonal hospitals, and subsequent rounds focused on public district hospitals. Within one year of training initiation (May 2004 - April 2005), provider coverage reached up to 60 facilities in 37 districts across Nepal.

Later, after successful pilot studies [16,21-27], training strategies and curricula were revised to include nurses and auxiliary nurse midwives and to incorporate medical and second-trimester abortion [28]. After its introduction in 2009, medical abortion coverage rapidly increased, eventually eclipsing the rate of MVA scale-up; establishment of MA services has been particularly successful in peripheral facilities, with 50% of primary health centers now having a listed provider for MA. By 2010 all 75 districts had at least one listed safe abortion site (with at least one qualified provider). This accomplishment is especially significant in light of challenges related to resource constraints, geography and a difficult political context. For example, the Maoist insurgency that peaked in 2004 resulted in many terrorist attacks and killings, in some cases seriously disrupting program activities, including training and supervision. Health service delivery was also frequently affected by strikes and transportation blockades that contributed to shortages of drugs and other commodities.

Instruction and support in **facilitative supervision **was an important factor in ensuring abortion services' quality and effectiveness. As part of whole-site orientation, for example, providers in management positions at authorized facilities learned to use performance improvement checklists during monitoring visits to audit staff performance and develop corrective action plans in such areas as patient counseling, infection prevention and post-abortion contraception. This participatory performance improvement approach fostered continual improvement in service delivery. Feedback from supervision visits offered immediate actionable inputs for staff, and site-based performance monitoring provided supervisors with important information on gaps in staff skills or knowledge, facility supplies and functioning, and staff motivation that could be communicated to policy-makers and leveraged for continued or additional support in training, funding or equipment for abortion facilities [29]. Another important aspect of abortion service implementation was emphasis on accurate and timely **monitoring **systems. With support from Ipas, the TCIC developed tools that were incorporated into existing health management information systems (HMIS) to measure the progress of abortion services and identify areas for improvement. On a monthly basis, all public-sector abortion care sites aggregate data on the number of postabortion care clients, induced abortion clients, clients accepting post-abortion contraceptives and clients with complications. Service statistics are reviewed monthly by site staff, quarterly at district review meetings with site facility managers, and annually at both regional and national review workshops. The Family Health Department also regularly reviews indicators on post-abortion complications and post-abortion contraceptive acceptance. If any district experiences more than a 2% rate of post-abortion complications or less than 60% post-abortion contraception uptake, the FHD helps identify challenges and plan counteractive measures. Thus, through routine monitoring and use of HMIS, all levels of health managers and stakeholders continually audit and improve the quality of abortion services.

#### Equipment and supplies

In Nepal, supplies for MVA, MA and second-trimester abortion are available through both public and private channels, with facilities able to use either system. Not surprisingly, sites with adequate financing--tertiary hospitals, larger health centers and private clinics - use private procurement systems, which typically are more efficient, while peripheral, rural and smaller health centers use the government system. The government is responsible for all upgrades to public-sector facilities and training centers, as well as for provision of basic health service equipment (not specific to abortion). Access to equipment, supplies and drugs, particularly MA drugs, has been complicated both by poor supply chain management and by over-the-counter, black-market sales along the Indian border. In addition, the Helms Amendment--a U.S. law banning use of foreign aid for abortion--has presented challenges for abortion supply logistics. Although USAID supports post-abortion care, which involves the use of MVA, USAID-funded programs cannot purchase MVA instruments - a restriction that has contributed to equipment shortages in Nepal [30].

#### Information

Partners have used a variety of information, education and communication (IEC) methods to generate knowledge about the availability of legal abortion in Nepal. One important strategy has been to engage various frontline volunteers in informing communities about and making timely referrals to safe abortion care. For example, with support from the Women, Children and Social Welfare Ministry, CREHPA's "Sumarga" project trained female community health volunteers (FCHVs) who act as grassroots health promoters to support poor and marginalized women by sharing information and providing financial subsidies and timely referrals for abortion services [31]. Here, too, the Helms Amendment has complicated matters, since FCHVs employed by USAID-funded NGOs are prohibited from incorporating safe abortion messages into their counseling services [32]. Ipas has also recently trained FCHVs in early pregnancy detection using urine testing kits and referrals for antenatal, contraception and abortion services [33-36]. In addition, Ipas and PSI have trained local pharmacists to provide women with knowledge about medical abortion, referrals to abortion services and information on indications for legal abortion in Nepal [37].

**Figure 2:**
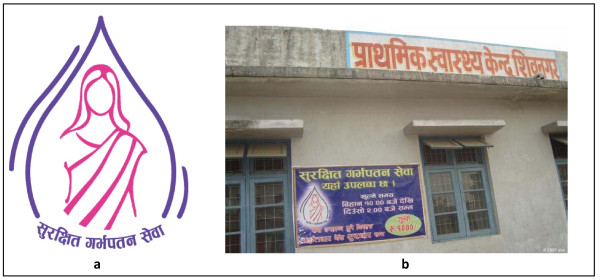
**Nepali Safe Abortion Logo, a) Original graphic and b) Applied in the field**.

A key innovation in Nepal was the TCIC's development and marketing of a safe abortion logo (Figure 2) - a visual symbol to designate facilities offering safe abortion services that is prominently displayed at all CAC sites. Through its extensive incorporation in safe abortion IEC materials and programs, the logo has become a widely recognized symbol of safe abortion in Nepal, particularly among vulnerable populations such as illiterate women. Partners have also used behavior change communication (BCC) strategies - including evidence-based community discussion groups and a serial radio drama [38,39]--to foster discussion about sexual health and to challenge commonly held negative beliefs and stigma around induced abortion.

### Positive impact

The early, coordinated, sustained and comprehensive planning and implementation efforts described above have led to remarkable achievements in the availability and use of safe abortion in Nepal. Comprehensive abortion care is now available in all 75 of Nepal's districts, many of which are in remote, mountainous areas, reflecting the government's emphasis on rapid decentralization.

As of December 2011, over 1500 health-care providers had been trained in safe abortion care, and 532 sites were authorized to provide safe abortion services (Table 1). Private facilities made up a significant portion of listed facilities at all levels of care: 139 (39%) of primary, 90 (53%) of secondary, and 6 (67%) of tertiary listed facilities are privately run. Over 500,000 Nepali women had received safe, legal abortion and contraceptive services through listed facilities since 2004 (Table 2).

**Table 1:** Preparation of Abortion Care Providers and Facilities, through December 2011

	Number
**Trained Providers**	

Physicians	881

Staff Nurses	371

ANMs	255

**TOTAL TRAINED PROVIDERS**	**1507**

**Listed Facilities**	

Primary	352

Secondary	171

Tertiary	9

**TOTAL LISTED FACILITIES**	**532**

**Table 2:** Women Served, January 2004 - June 2011*

Year	Public	MSI	FPAN	Total
January 2004-June 2004	719	---	---	719

July 2004 - June 2005	5639	3076	1846	10561

July 2005 - June 2006	9267	34518	3666	47451

July 2006 - June 2007	9416	57625	6433	73474

July 2007 - June 2008	21859	67426	8093	97378

July 2008 - June 2009				83978

July 2009 - June 2010				88938

July 2010 - June 2011				95,305

**TOTAL**				**497,804**

*Ipas site-wise monitoring was conducted from 2004 - 2008. Beginning in 2009, abortion service monitoring was conducted under the national HMIS system (reported district-wise) and cannot be disaggregated by facility type. In addition, the HMIS-reported number is likely an underestimate of actual women served

MSI=Marie Stopes International and FPAN = Family Planning Association of Nepal

**Figure 3:**
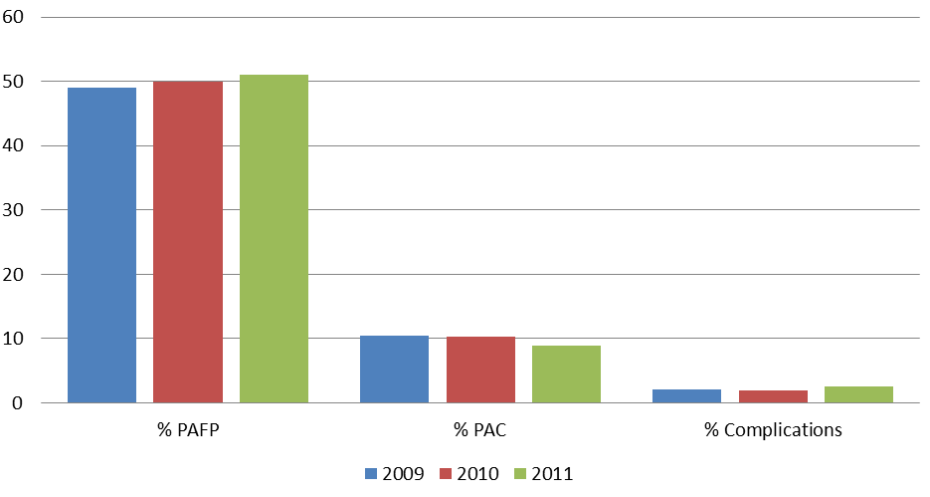
**Key Abortion Care Indicators from HMIS System, 2009-2011**. (PAFP = postabortion family planning, PAC = postabortion care).

Since integration of safe abortion service monitoring into the national HMIS system in 2009, four main indicators have been followed: number of women served, percentage of women receiving postabortion care, percentage of cases with complications, and percentage of women receiving postabortion contraception. The number of women served has increased by year, for totals of 83,978, 88,938 and 95,305 in 2009, 2010 and 2011, respectively. However, the other indicators remained fairly stable during the three-year time period: 50% of women receive postabortion contraception, 10% of all abortion-care services delivered is for postabortion care, and 2% of women experience complications (Figure 3).

Partners have also worked to ensure that services are safe and of high quality. A 2008 study found that of 7,007 abortion clients selected from a sample of facilities across Nepal, only 2% (n = 131) experienced complications following the procedure, and all those women were treated at the facility where they had their initial abortion [40]. In addition, the percentage of abortion complications at essential obstetric care facilities dropped significantly to 28% from 54% of all complications in 1998 [41,42]. A 2008/2009 study on eight Safe Motherhood districts also revealed, however, that the percentage of facility deaths due to abortion increased slightly from 10% in 1998 to 14% in 2008/09 [41]. Both indicators may reflect increased access to health care services as well as increased willingness to seek such care; before legal reform, women who suffered even serious complications from abortion were unlikely to seek care at a hospital for fear of imprisonment if they did survive.

Along with other Safe Motherhood efforts, the large-scale provision and utilization of safe abortion services has contributed to substantial declines in maternal mortality in Nepal. The estimated maternal mortality ratio dropped from 539 in 1996 to 281 and 229 in more recent studies from 2006 and 2009 [41,43].

### Lessons learned and ongoing challenges

Nepal owes its relatively quick success in making safe abortion widely available after reform of its restrictive law to several elements of planning and implementation, which may offer useful guidance to other countries.

First, all efforts in planning and implementation (as well as advocacy leading to legal reform) **involved a diverse partnership led by government **with members from international and local NGOs, advocacy groups and private partners. Inclusion of an array of partners ensured that the entire process was guided by a wide range of expertise and--along with mutual, transparent decision-making--helped achieve broad-based buy-in to a comprehensive approach. Unified under the common banner of preventing deaths and injuries of women from unsafe abortion, partners put the interests of Nepali women ahead of interpersonal and organizational differences and maintained a professional and collaborative spirit during the course of working together. The **commitment and leadership of the Nepali government **in coordinating the different players has been pivotal.

Another important foundation of success has been **integration of abortion care with the national Safe Motherhood initiative and with the broader health system**. The involvement of staff nurses and ANMs as service providers and FHCVs for information and referrals facilitated efforts to integrate safe abortion into the existing health system network, which included the use of existing HMIS to assist monitoring and evaluation efforts.

**Policymakers' reliance on public-health evidence **- from other countries as well as in Nepal itself - has helped ensure that Nepali women benefit as fully as possible from scientific progress and that legal abortion services meet the highest international standards. Examples include the adoption of medical abortion and modern techniques for second-trimester abortion. The evidence-based inclusion of staff nurses and auxiliary nurse midwives as providers of first-trimester abortion care has been especially critical in making care widely available, in contrast to settings such as India, where even first-trimester abortion has remained the purview of a small number of highly trained physicians. Nepal's more inclusive approach has greatly increased the reach of services in terms of numbers of trained providers and geographic coverage. Continued reliance on public-health evidence will be essential to further program success.

Despite these significant benefits, essential needs remain. Foremost among these is **continued expansion of safe abortion service delivery sites **for even broader geographic reach, since many women - especially in rural and mountainous areas--still lack ready access. In some districts, safe abortion services are still limited to district hospitals which are a three- or four-day walk from some villages.

Expansion of safe abortion services has been hampered by persistent **training and staffing challenges**. For example, the number of training centers is insufficient to meet the high demand for trained providers, and many providers, particularly those at public facilities in underserved areas, have difficulty getting work release to attend trainings. Hospitals and clinics operating as training centers are also burdened by the dual demands of training and regular service provision. Furthermore, high staff turnover, particularly in remote facilities, creates significant service gaps in care for the most vulnerable women. The dearth of trained providers and authorized clinics in some areas can cause grievous delays for women, preventing some in later stages of pregnancy from obtaining services within the legal timeframe.

Challenges also remain in providing **access to affordable services for poor women**. As noted, fees for induced abortion are not uniform; they can be prohibitively high in some public facilities and include hidden costs for drugs, materials and equipment. In addition, few women are aware of eligibility requirements for subsidies. A Nepal Supreme Court decision in February 2011 (Lakshmi Dhikta v. Nepal) [44], brought on behalf of a rural woman who was unable to afford an abortion, reaffirmed Nepali women's right to reproductive choice and emphasized the government's obligation to guarantee access to affordable safe abortion services for all women, but the government has not yet revised fee policies accordingly.

As suggested above, the U.S. government's **Helms Amendment **hinders full implementation of nationwide safe abortion services and full integration of this care into other elements of maternal health care by restricting use of resources associated with USAID-funded programs [45]. While the recent reduction of USAID support to public family planning clinics in Nepal has alleviated this problem somewhat, it remains an important challenge.

As with many health services, **obtaining accurate, complete monitoring data **on abortion services has also been challenging in Nepal. Often, the provider responsible for completing the logbook is over-burdened and unable or unwilling to enter complete patient data. Moreover, private facilities have no reporting obligations, making their monitoring data unavailable to the government. Practitioners and partners also need impact evaluation data to measure the long-term effect of abortion provision on reducing unsafe abortion and related morbidity and mortality.

In addition, while all stakeholders agree that **preventing repeat abortions **requires further strengthening of contraceptive services, especially in peripheral health facilities, there is disagreement on the approach needed. The Family Health Division prefers to emphasize long-term and permanent contraceptive methods when counseling postabortion clients, while members of the Technical Committee for Implementation of Comprehensive Abortion Care want to ensure that women can freely choose from a full range of contraceptive options. They recommend overall strengthening of contraceptive services, along with better integration with abortion care, both to improve women's access to comprehensive reproductive health care and to reduce the stigma surrounding abortion. Evidence of high suicide rates among young women [41] and the strong social disapproval of unwanted pregnancy among unmarried women [45] further supports the need to address abortion stigma, in communities and among providers. In addition, at many public health facilities, abortion clients are subject to long waiting times while patients with conditions considered more urgent are attended to, indicating that abortion is not considered a priority service.

Finally, policymakers and program managers face challenges related to the practice of sex-selective abortion, which the law prohibits but which, as in neighboring India, remains an issue of great concern societally and for health-care providers [46,47]. Policymakers have recently recognized the need to strengthen monitoring systems to ensure compliance with the law while also addressing deep-rooted issues such as gender inequity that contribute to the practice. In implementing this two-pronged strategy, it will be important to maintain a strong focus on protecting women's health and rights.

### Recommendations

Possible steps to address these and related challenges include the following:

• **Safeguard the simplicity of the facility certification process**. Stakeholder vigilance is needed to preserve the process's integrity and to ensure expansion of service availability across the country.

• **Ensure that abortion services are affordable for all women**. By eliminating the cost distinction between abortion and other elements of maternal health care, the government can facilitate access to critical care for many women and help diminish the stigma surrounding abortion. At a minimum, the existing policy on subsidies for poor women needs to be clarified and widely disseminated, both to health-care facilities and to women. Advertising the fee waiver through a variety of media could help educate women about their rights and empower eligible women to ask for it.

• **Incorporate pre-service CAC training into curricula of medical, nursing and midwifery schools**. Institutionalization of pre-service education in comprehensive abortion care would alleviate the burden on training in functioning health facilities and ensure the ongoing availability of a large pool of trained providers. It would also combine the once segregated postabortion care and induced abortion training.

• **Expand training of community-based health-care providers**. As mentioned, auxiliary nurse midwives have been trained to provide safe abortion services, and FCHVs to disseminate information to communities. To enhance outreach and access to care at the community level, training of such frontline workers should be scaled up, particularly in rural and remote areas, and perhaps expanded to encompass additional areas of abortion service provision; more research--for example, pilot training of FCHVs in screening women for medical abortion eligibility--is needed to determine how best to expand such roles.

• **Strengthen referral links between broader reproductive health care, including contraceptive services, and comprehensive abortion care**. Better engagement of frontline workers and service providers could help strengthen referral links both to direct women seeking pregnancy termination to registered abortion-care sites and to ensure that they receive post-abortion contraception and other critical services. Improved, timely referrals can help counteract late detection of pregnancy, which prevents some women from obtaining safe abortions. Linking clients to appropriate post-abortion contraception services and providing adequate follow-up care for contraceptive continuation is also crucial to ensuring that women in Nepal can fully exercise their reproductive choices.

• **Continue innovative information campaigns**. To educate the public, increase awareness of the new law and of the availability of safe abortion, and to decrease stigma associated with abortion, programs should continue to employ effective methods of behavior change communication. Messaging efforts should take into account the specific contextual and language needs of vulnerable populations such as those with low or no literacy, ethnic minorities and those living in remote regions.

• **Educate policymakers, program managers and providers about Helms Amendment restrictions to minimize harmful over-interpretation**. Regular updates on the national safe abortion policywould help these stakeholders understand the extent of the Nepali government's budgetary and other support for elements of CAC such as MVA and contraceptive supplies, postabortion care training and staff salaries, and reduce their fears about jeopardizing donor funding.

After a decade of legal abortion in Nepal, it is also clear that **further changes are needed in the abortion law **to eliminate existing constraints and more fully address women's needs. Specifically, the current 18-week limit for women and girls who experience rape or incest provides insufficient time for many to access legal abortion. In many instances, they are subject to family and societal pressure to keep their pregnancy secret, pushing those who ultimately decide to seek abortion beyond the legal limit. The current restriction also fails to take into account issues relevant to all women facing unintended pregnancies, such as delay in recognizing pregnancy; stigma and other factors that may delay their decision to have an abortion once they know they are pregnant; and lack of knowledge about the availability of legal abortion. Although the mental health indication allows some scope for providers' interpretation, its application is variable and thus unfair. Making safe legal abortion available at any time during pregnancy for women and girls who have experienced rape and incest would more realistically - and more compassionately--reflect the realities of their lives. Efforts are fortunately underway to draft a new law that would address these concerns.

## Conclusion

Nepal's experience introducing and scaling up safe abortion suggests important lessons for other countries seeking to reduce maternal mortality and morbidity from unsafe abortion. Comprehensive, systematic planning and effective coordination among a range of stakeholders led to remarkably rapid and successful implementation of safe abortion services, with a strong positive impact on public health. Strong government commitment and leadership has been vital to the acceptability and sustainability of comprehensive abortion care services. Furthermore, program and policy decisions were based on sound evidence, including experience in other settings and successful pilot efforts. Finally, use of a systems approach to integrating safe abortion training and service delivery helped ensure that at least half a million Nepali women have been able to benefit from the availability of safe legal abortion services, linked to contraceptive counseling and services to help them prevent future unintended pregnancies, within a short time after the law changed. This important progress has significant implications for the health and well-being of Nepali women, families and communities.

## Competing interests

The authors declare that they have no competing interests.

## Authors' contributions

All authors contributed to conceptualization of the review and to editing the paper. KA contributed to research, methodological design, data analysis and editing. IB, AH and KA contributed to program design, implementation and evaluation. GS contributed to the research, data analysis and writing. All authors have read and approved the final version of the paper.
